# Parasitology of the twenty-first century: are we moving in the right direction?

**DOI:** 10.1099/jmm.0.002064

**Published:** 2025-09-10

**Authors:** Kinga Kowalewska-­Grochowska, Romina Reyes, Pauline Tomlin

**Affiliations:** 1 University of Alberta, Edmonton, Alberta, Canada; 2 Alberta Precision Laboratories Public Health Lab, Edmonton, Alberta, Canada; 3 LifeLabs, Burnaby, BC, Canada; 4 University of British Columbia, Vancouver, BC, Canada

**Keywords:** artificial intelligence, diagnostic parasitology, globalization effect, specialized expertise, workforce projections

## Abstract

For thousands of years, parasitic infections have represented a constant challenge to human health. Despite constant progress in science and medicine, the challenge has remained mostly unchanged over the years, partly due to the vast complexity of the host–parasite–environment relationships. Over the last century, our approaches to these challenges have evolved through considerable advances in science and technology, offering new and better solutions. Unfortunately, in the twenty-first century, this diagnostic evolution was suddenly confronted with a dramatic change of biological relationships, never witnessed in history before the uncontrolled expansion of the human population, globalization and hyperconnectivity technology have exerted a massive socioeconomic impact on individuals, communities and the environment, sending a ripple effect throughout the world of parasites. Urbanization, pollution and the unsustainable exploitation of natural resources have caused shifts in biomass and the fragmentation of habitats, leading to the movement of parasites into new hosts and territories. At the same time, changes in human population structure and distributions due to armed conflict and poverty created massive migration of entire nations and communities, resulting in the redistribution of parasitic diseases. To make the situation worse, the population of many receiving countries of North America and Europe is ageing, leading to a critical shortage of a specialized workforce essential to deal with the new diagnostic challenges. Unfortunately, this vicious circle is not yet apparent to all. The highly specialized field of parasitology is at a particular risk for such major crises in the near future. Heightened awareness of such risks is an essential step to start discussions and planning to mitigate these very real health threats.

## Introduction

Parasitology is a biological discipline focusing on parasites (protozoa, helminths and arthropods), their hosts and the relationships between them. Medical parasitology concentrates on parasites of humans and their impact on human health.

Its scope is determined by a parasitic way of life rather than the organism itself or the environment in question. As such, it draws on many other disciplines such as cell biology, immunology, genetics, evolution and ecology, to name a few. Medically, parasitic infections do not always manifest as clinical illnesses, and in many cases, there is a blurred line between parasitism and commensalism, adding to the complexity of the host–parasite–environment relationships.

The story of human parasitology is rich and convoluted, yet often perceived as less immediately relevant to human welfare than bacteria and viruses, known to create devastating epidemics that changed the course of history.

And yet, parasitism is (and always was) an intrinsic attribute of life. It has been demonstrated in every existing species since the beginning of life on Earth, ca. 4.4–3.8 billion years ago [1]. Our present-day parasites are the consequences of diverse associations between life forms, from molecules to plants and animals, following the path of co-evolution within a vast host–parasite environment [2].

These associations continued with the arrival of humans. Parasitic remnants have been found in coprolites and mummified human remains dating as far back as 1.5 million years [3,4].

Not surprisingly, parasites seem to be the most successful beings on our planet. Currently, they constitute at least half of all living species [5] and up to 78% of food web links [6]; they coexist, co-evolve and persist alongside their host sponsors, exercising lifestyles that represent one of the most successful consumer strategies on Earth.

The human body is known to house about 300 helminth and 70 protozoan parasite species with an ancestral or zoonotic origin; they often live in uneasy harmony with each other, despite the fact that some of these pathogens can cause serious health consequences across the globe [7].

Parasites can cause a wide range of illnesses in humans and animals; they can be transmitted through contaminated food and water, as well as person-to-person or animal contact. Accurate and timely diagnosis allows for appropriate treatment of those infected, prevents further transmission and is vital for public health surveillance and intervention programmes to contain risks and control further spread.

Despite all efforts, parasitism remains a constant and unrelenting challenge to mankind, even though approaches to addressing this challenge have evolved over the years.

In this review, we will address the evolution of these challenges, our attempts to solve them, and discuss future directions and potential threats.

## The diagnostic evolution

The first significant step towards the detection of parasitic agents of human disease was microscopy – a tool that remains a diagnostic gold standard to this day.

The history of the microscope dates to 1590 when Dutch father-and-son team Hans and Zacharias Janssen created the first microscope. The Janssen instrument was then used by Robert Hooke to study minute objects, later illustrated in his book *Micrographia* in 1665 [8]. However, the true birth of microscopy (and microbiology) was ascribed to Anton Van Leeuwenhoek, a Dutch cloth merchant who used the microscope to study the quality of his wares during a visit to London in 1666. Upon returning home, Van Leeuwenhoek started constructing simple microscopes to continue his investigations. As a result, he discovered tiny objects, ‘animalcules’, which he reported to the Royal Society in London in 1673 [9].

Van Leeuwenhoek’s discovery of protists and bacteria defied contemporary belief in spontaneous generation of life and diseases and earned him a position as the father of microbiology (not to mention selling 500 of his microscopes!) [10].

Microscopy thrived in parasitology for years. It covered examination of simple wet mounts, later supplemented by basic stains (iron haematoxylin or trichrome for stools, Wright and Giemsa for blood and tissue parasites etc.), and was soon followed by targeted Ziehl Neelsen and Kinyoun stains for acid-fast organisms, as well as non-specific fluorescent stains, like acridine orange or calcofluor white.

These stains were relatively cheap and easy, and the methodology was accessible to all laboratorians, as the microscopy established itself as a basic tool for diagnosis.

However, this technique was labour- and time-intensive and required not only expertise in reading and interpretation of slides, but also submission of clinically appropriate biological material (often scarce or not easily available), as well as the presence of intact parasite forms.

The next step in diagnostic evolution had to address the challenges of morphology-based approaches. The new strategy—immunodiagnostics—focused on parasite antigens and/or host antibodies produced in response to infection, rather than their visualization.

Many methodologies were developed over the years: haemagglutination, complement fixation tests, ELISA, indirect/direct immunofluorescent antibody assays, immunoblotting and then newer techniques such as luciferase immunoprecipitation system, among others [11,13].

The antigen detection techniques covered selected pathogens such as *Plasmodium* spp*.*; *Wuchereria bancrofti* [14]; gastrointestinal protozoa, including *Cryptosporidium*, *Giardia* and *E. histolytica* [14]; and even *Cyclospora* sp. All could be performed directly on randomly collected stools or other patient specimens.

Serology – the other arm of immunodiagnostic testing – targeted host response to infection; a vast array of antibody assays (both commercial and lab developed) covered numerous parasitic agents and diseases and made such assays a popular and convenient diagnostic tool [12,15].

Both types of immunodiagnostic tests were invaluable in situations where a trained eye was not available and appropriate specimens were hard to come by; they could be performed on blood or stool—both sample types that are easy to collect at most times. They offered increased sensitivity when compared to microscopy and the possibility for automation.

Immunodiagnostic methods flourished over the years and are still in use to this day, even though the landscape of parasitology continued to evolve and create a need for new approaches.

One such reality – economic pressure – recognized the need to simplify diagnosis and decrease costs; thus, the lateral flow assay (LFA), a rapid and simple paper-based analytical platform, was developed and successfully implemented in many diagnostic laboratories in the 1990s.

Although LFA remained in use for decades, it was the COVID-19 pandemic that forced a dramatic revival of these assays in all healthcare settings (and as over-the-counter diagnostic solutions) [13].

As financial and human resource constraints tightened their grip on diagnostic parasitology, these old LFAs gained new traction as fast, simple and cost-effective point-of-care tests targeting the commonest parasitic infections: giardiasis, cryptosporidiosis and amebiasis [16,17].

However, these early non-morphological methods came with their challenges: cross reactions between parasite species leading to false positives and false negatives, co-infections, unreliability (and variability) of serological responses, and lack of standardization between assays [13].

Ultimately, it was the meteoric rise of molecular biology and its foray into diagnostics that changed the landscape of parasitology forever. Many challenges of microscopy and serology could now be overcome since nucleic acid amplification tests (NAAT) offered much greater sensitivity and specificity. Traditional PCR and nested PCR were soon followed by reverse transcription PCR for many parasites, with multiplexed PCRs able to detect multiple sequences in the same reaction tube and thus diagnose multiple infections simultaneously.

Other molecular platforms emerged in short succession, such as loop-mediated isothermal amplification, Luminex-based assays, multiplex ligation-dependent probe amplification and digital PCR, followed by DNA sequencing [11,15, 18].

Utilizing these new technologies, parasitology diagnostics began leveraging new approaches, already in use for other infectious diseases: syndromic panel testing, allowing more focused and integrated workflows, and replacing routine bench work and expertise.

These molecular panels were able to include and detect a variety of viral, bacterial, fungal and parasitic pathogens, typically focusing on a single clinical syndrome such as gastrointestinal infections. While the molecular targets on most multiplex gastrointestinal (GI) panels include an extensive list of bacterial targets, the parasitic ones are limited to only a few (*Giardia lamblia*, *Cryptosporidium* spp., *Entamoeba histolytica* and *Cyclospora cayetanensis*) [19].

As a screen, these panels work well to cover the most common agents of parasitic GI infections. However, they do not detect other parasitic species that are also clinically important and highly prevalent in certain populations.

The diagnostic evolution continued with the arrival of the high-throughput metagenomic next-generation sequencing, allowing sequencing of the entire human genome within a day. This technology has had a transformative effect on our understanding of parasite genomics and diseases they cause [20,21] and is highly likely to spill over to diagnostic parasitology in the near future.

Although not yet fully adapted to clinical settings, several different platforms have appeared on the scene, heralding the era of proteomics: matrix-assisted and surface-enhanced laser desorption ionization time-of-flight mass spectrometry or liquid chromatography combined with mass spectrometry. These techniques led to the discovery of potential biomarkers and their distinctive proteomic fingerprinting patterns that will help design diagnostic tests for parasitic diseases and assessment of cure in the future [11].

## The challenges of an uncertain future

Entering the third decade of the twenty-first century, these new developments brought seemingly endless and exciting opportunities for parasitology diagnostics.

However, the very same twenty-first century has experienced a dramatic change, unparalleled by any previous events in the history of humanity. Globalization and hyperconnectivity of rapidly expanding information technology exerted a massive socioeconomic impact on our communities and individuals (including the workforce), as well as on our environment and quality of life. Gradually, these changes exert a ripple effect throughout the world of parasites – their biology, ecology and relationships with hosts. The variables and consequences are not fully known, but some are noticeable even now.

With the uncontrolled expansion of the human population and the industrial revolution in the Anthropocene, the Earth’s environment has gradually and irreversibly changed. These changes were disproportionately accelerated in recent years. Global temperatures increased over 1° compared to the pre-industrial era (1880s) and alterations in patterns and distribution of rainfall brought on floods and droughts, wildfires and other extreme weather events.

Urbanization, unsustainable exploitation of natural resources for industry and agriculture caused shifts in biomass and fragmentation of habitats. Increased connectivity between humans and animals on a global scale led to the movement of parasites into new hosts and territories and many other untoward spillover events [22]. These changes triggered a cascade of climate-adaptive development in parasites, influencing a range of host–pathogen interactions [23].

The patterns of parasitic diseases in humans and animals changed, gradually increasing the parasite global footprint and redistributing their ecological niches. This has already manifested itself in several settings, often with alarming consequences. Examples include water stress caused by climate events and aggravated by building dams or redirecting water for irrigation systems; this affects populations of snails (intermediate hosts for Fasciola and Schistosoma) and, ultimately, distribution of human infection.

There are many other examples, such as outbreaks of intestinal protozoan infections after heavy rainfalls overwhelming wastewater systems, re-distribution of soil-transmitted helminth infections due to droughts or floods or even northern expansion of tick habitats caused by rising temperatures [24,25]. As a result, we are experiencing a gradually developing shift in ecological reality. Almost imperceptibly, over the years, this abundance of changes created a brand-new global parasitology landscape, one that we are only now reluctantly acknowledging.

But these changes are not limited to the parasites. The human population structure is changing too, both in terms of numbers as well as distribution. These changes affect not only countries relying on immigration for growth, like Canada, but also historically well-established European states. Not surprisingly, a large part of these population shifts is driven by the re-emergence of an age-old factor: human conflict. Interestingly, one of the earliest accounts of the connection between wars and (parasitic) infections was published in 1918 [26].

**Fig. 1.**
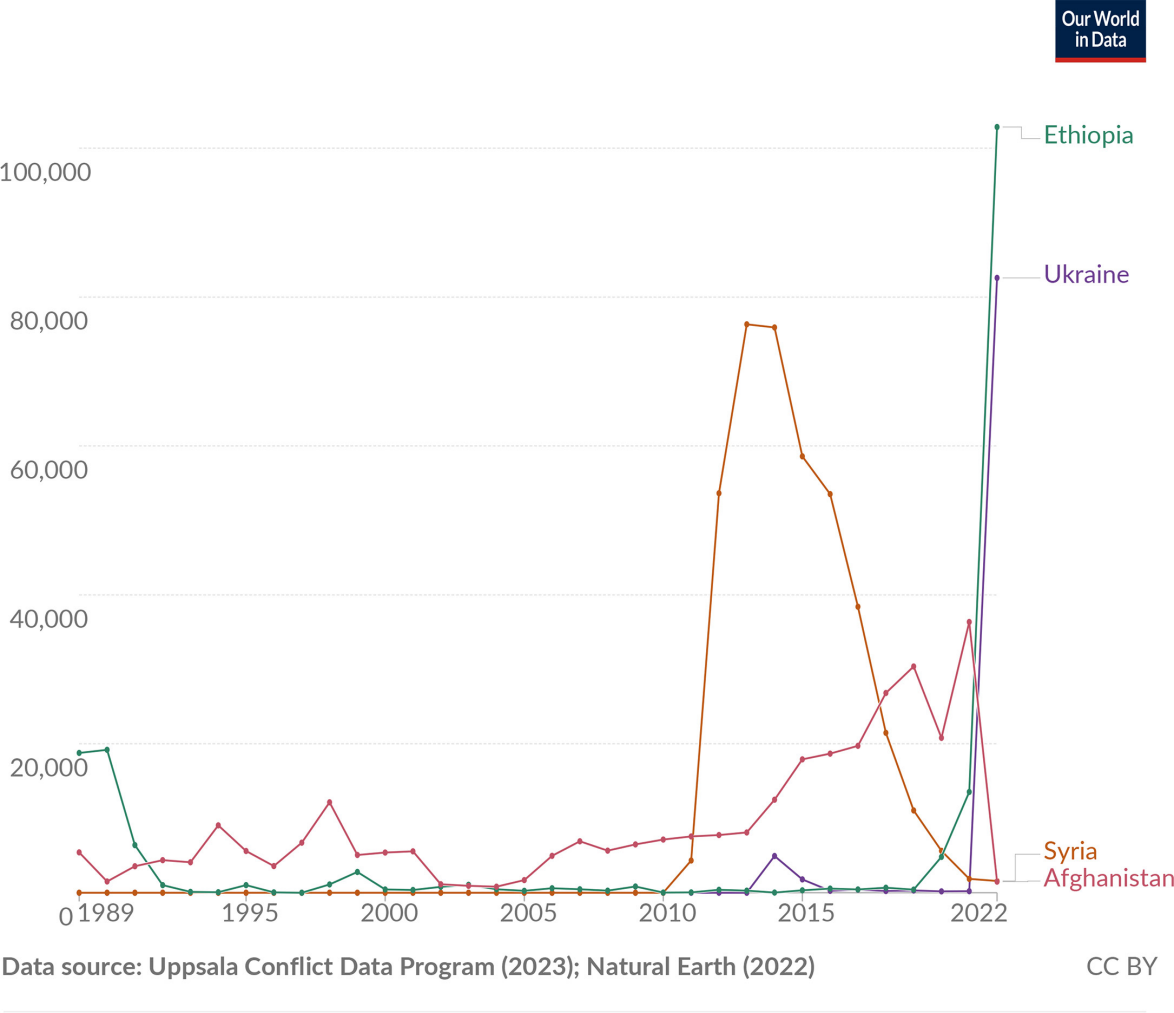
Deaths in armed conflicts based on where they occurred. Included are deaths of combatants and civilians due to fighting in armed conflicts¹ that were ongoing that year.

Overall death toll from active fighting in wars amounts to about 37 million since the 1800s (not including those who died of war-related hunger and disease). After the massive killing peaks of both World Wars, our planet entered a period of relative stability, which extended to around 2010, only to increase exponentially again (see Fig. 1, from Uppsala conflict data programme, 2023) [27,28].

Prolonged and intractable conflicts, aggravated by climate change, affect many of the already fragile states and regions and curtail resources available for the people. Poverty, political instability and even starvation created a new phenomenon – mass migration of entire populations in search of food and personal safety [29].

Worldwide, there were 281 million international migrants (all causes) in 2020, amounting to 3.6% of the world’s population that year, according to the United Nations’ International Organization for Migration [30]. Worldwide escalation of armed conflicts created a new entity – forcibly displaced peoples, estimated by the United Nations Refugee Agency at 122.6 million in mid-2024 alone.

Canada is planning to accept up to 500,000 new immigrants annually in the coming years, according to Immigration, Refugees and Citizenship Canada’s new Immigration Levels Plan. This number represents the highest levels in Canadian history and does not even include incoming undocumented migrants [31].

All this gradually changes the very fabric of our society, and Canada (already an ethnic mosaic, just as many other European countries) is gradually becoming a vastly different nation.

This new structure of a twenty-first-century society generates brand-new concerns and challenges. The sheer volume of migration brings along new diseases, including new parasites. Previously considered unusual, these parasites are now becoming a part of mainstream health concerns, bringing in infections that are more difficult to diagnose.

This raises a very important question: are current human resources in European and North American laboratories sufficient to handle the needs of the incoming population that is growing in numbers as well as in complexity of their medical issues, such as parasitology?

**Fig. 2.**
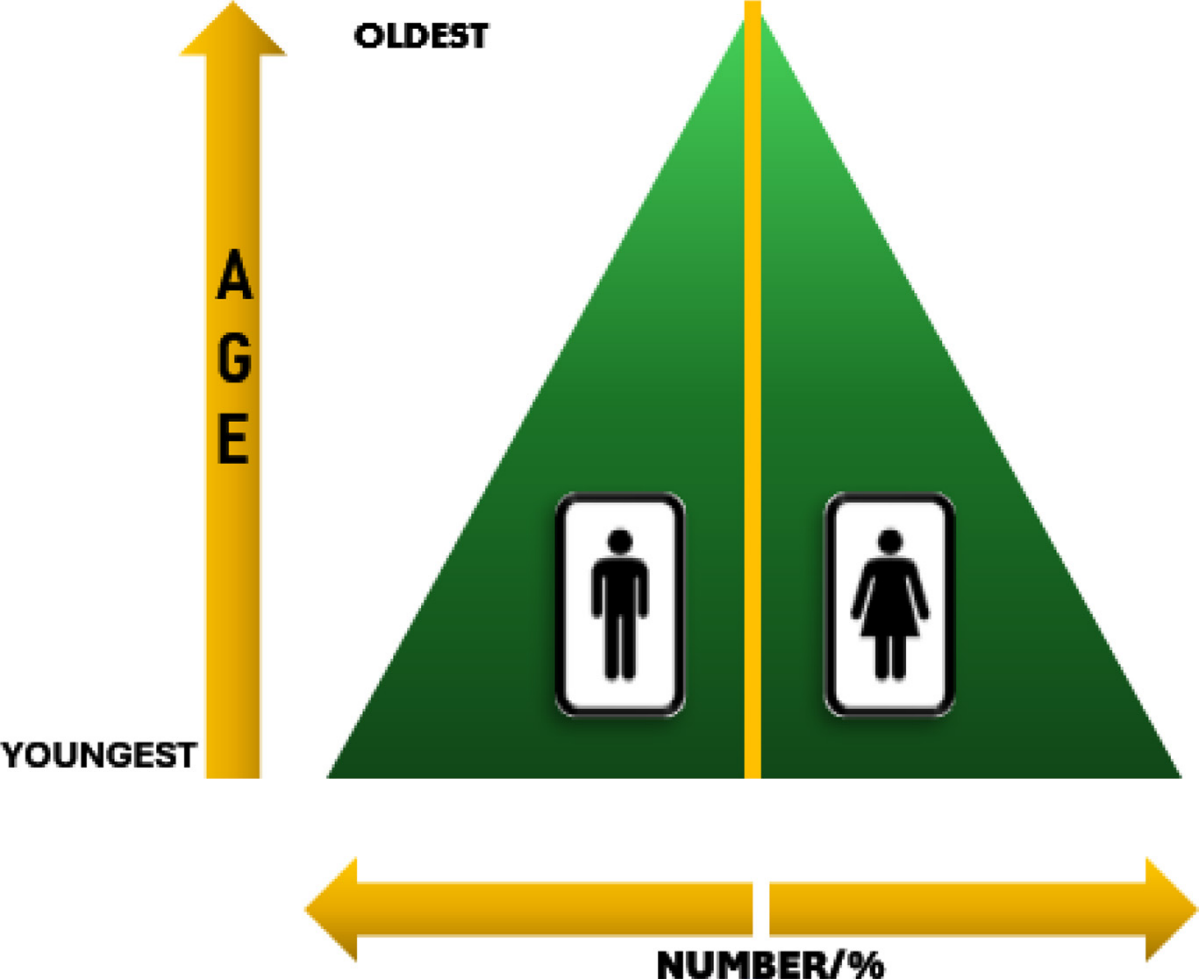
Standard population pyramid.

The answer to this question paints a rather bleak picture. Canada (along with many other Western countries) is ageing. Its population pyramid (or age structure diagram – a graphical depiction of overall age distribution of a given population) has changed since the postwar era: from ‘broad-based pyramid’ (Fig. 2), indicating high proportion of young people, to ‘inverted pyramid’, with decrease in the younger age groups and increase in population at or above retirement age. This phenomenon is caused by fertility rates dropping below replacement levels as well as increasing life expectancy. The current population changes create an imbalance between needs and fulfilment. The *increasing* elderly population with higher health needs (who have already left the workforce) requires more medical (and social) support; this support, however, can only be provided by a steadily *decreasing* pool of young people entering the labour market (including healthcare and diagnostic labs). This creates an imbalance between supply and demand, particularly in the context of a very specialized area such as diagnostic parasitology, heavily reliant on human knowledge and expertise [32,33].

A downstream effect of this imbalance is an ongoing critical shortage in the workforce, particularly in areas requiring specialized knowledge that takes years to develop and maintain. As the older Canadians leave the job market due to burnout, retirement or health challenges, the replacements are just not around.

Between 1950 and 2023, the number of people over 65 (leaving the workforce) increased from 7.5 to 21.3% of the population, while the population of *future* workers (15 and under) decreased from 29.4 to 15.4%. This disturbing disproportion is likely to persist, and immigration may not necessarily provide the immediate relief needed to support social spending on education, health or eldercare [34][Bibr R33]

**Fig. 3.**
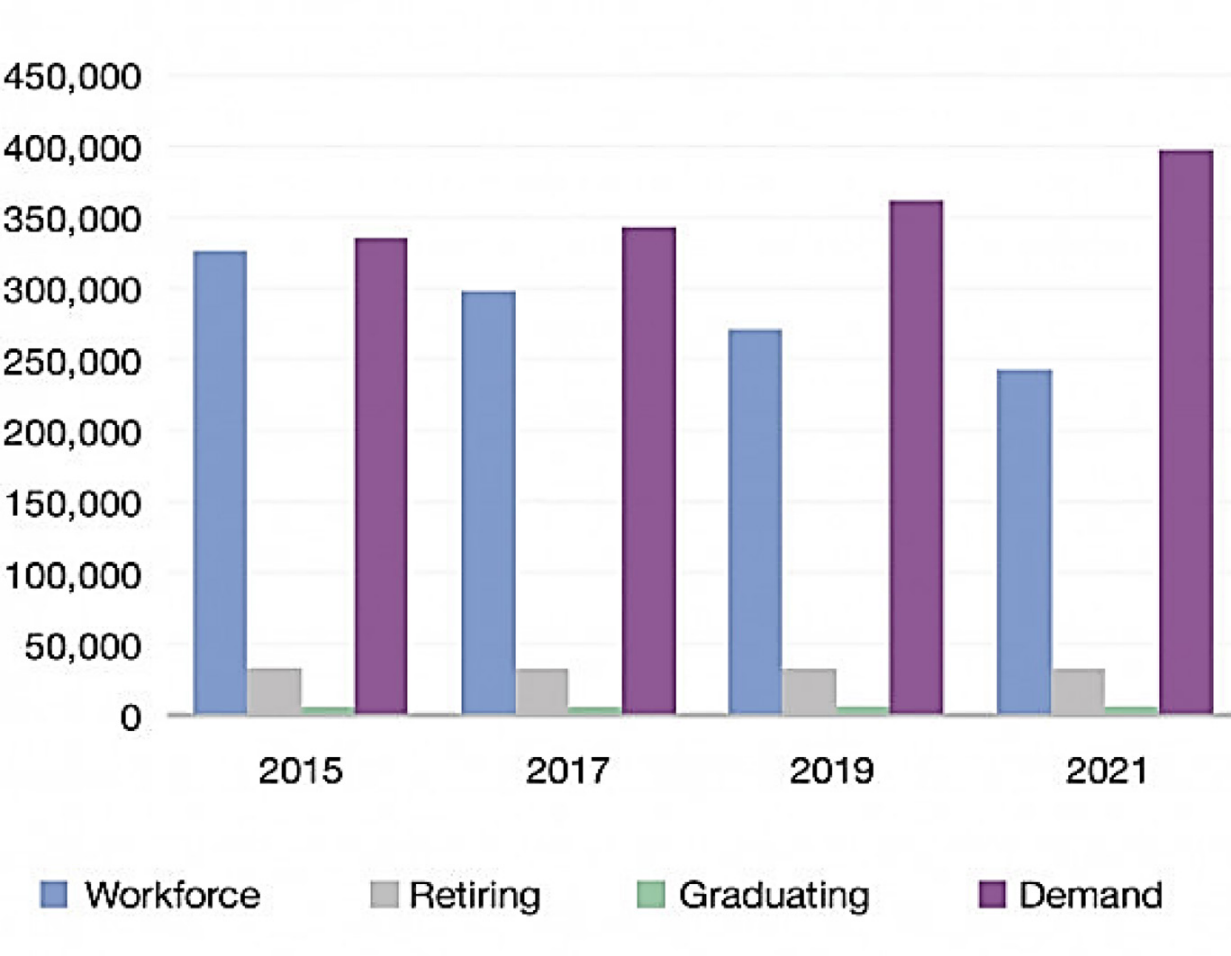
CLS workforce projection. *From: Laboratory Staffing and a Faltering Connection, Medical Lab Management, December 2017-Vol. 6 No.10* [35].

This is particularly poignant in laboratory medicine. The 2017 data from the US Department of Labor and Statistics (Fig. 3) indicated a projected workforce shortage of over 150,000 clinical laboratory science professionals in the USA (almost 40% of the total workforce demand deficit) [35]. This has been noted in several healthcare settings in Canada and beyond.

The current situation brings up a lot of concerns. In addition to expanding (and changing) patient populations, we are facing critically negative shifts in the workforce, particularly those with specialized skills. This holds for any medical specialty, but parasitology is particularly vulnerable. For several reasons, real and perceived, it remained a part art/part science specialty, where a morphological image interpreted by a trained human eye cannot be confirmed by an objective tool such as biochemical reaction, culture or even molecular methodology.

In theory, the advent of molecular diagnostics should provide solutions to the loss of human expertise.

However, as enticing as it may be, non-morphology-based diagnostic tests come with limitations.

For example, a specimen may be incompatible for molecular testing due to inhibitory/interfering substances or because of inappropriate collection barriers that may not be easy to overcome or control.

But most importantly, currently available assays do *not* cover everything. Humans can harbour hundreds of parasite species, of which about 90 are common. Of these, only a few are covered by serology, antigen detection or even NAATs, due to inadequacy of sequence reference databases.

This becomes particularly important when considering greater heterogeneity of North American and European populations – an increasing challenge from both clinical and epidemiological points of view. The current scope of testing does not encompass unusual parasitic entities, now arriving at the forefront of healthcare. Syndromic testing, be it lateral flow or multiplex PCRs, geared towards ‘common’ pathogens of yesteryear, slowly becomes less relevant [36].

Furthermore, both serology and NAAT do not distinguish active from past infections due to the persistence of antibodies or parasitic DNA. Both cannot differentiate viable from non-viable forms, so false-positive PCR results can continue for extended periods post-treatment or recovery, representing the remnants of free pathogen molecules that do not signify ongoing infection [36,37].

As new NAAT experience is gained, the interpretation of these supersensitive tests starts raising some new questions: what is the true clinical significance of results? What do they say about the disease, or its public health consequences?

Even looking towards new research, overreliance on molecular technology can (perhaps counterintuitively) hinder the discovery of new species, because morphological characterization is still required for a valid description of any new parasitic species (even if DNA sequences have already been derived).

With that in mind, it is worth revisiting the broad universality of microscopy as it contrasts with the targeted nature of NAATs. While it is time-consuming, observer-dependent, and not as sensitive as other options, the role and focus of microscopy remain pivotal because of its broad scope. The closest analogy is quite evident in bacteriology, where a 140-year-old Gram stain successfully coexists with next-generation sequencing.

It seems, therefore, clear that, in parasitology, molecular methods should *complement* rather than replace old-school morphological assessment. Contrary to developing trends, preserving such a ‘wide-angle lens’ approach to parasite diagnostics is particularly appropriate in the new era of diversity of challenges, brought upon by our changing world.

At this point, it is important to bring up one more issue – a new obstacle to address: the rapid decline of specialized workforce and the disappearance of expertise that takes years to develop.

## The role of artificial intelligence

The loss of human expertise is thought to be resolved by artificial intelligence (AI). In a 2019 survey of 487 pathologists practising in 54 countries, 80% of respondents predicted the introduction of AI in the laboratory within the next 10 years [38]. And yet, not many of us truly understand the nature and challenges of the AI itself.

According to the International Organization for Standardization, AI is ‘a technical and scientific field devoted to the engineered system that generates outputs such as content, forecasts, recommendations or decisions for a given set of human-defined objectives’[39] .

In plain words, AI is a simulation of human intelligence processes by a computer – basically, a tool to analyse large amounts of data and make predictions or decisions while continuously improving its performance over time. To fully appreciate its complexity as well as risks and benefits, let’s briefly unpack AI’s core mechanisms.

The first step is the extraction of raw information from various sources via data pipelines and transfer to a data repository. But this is not as simple as it sounds.

Each day, quintillions of data are generated across the world, and this massive amount of information needs to undergo some processing to create a basis for further analysis. For this to happen, computers need mathematical algorithms or modelling. AI provides these bases, custom-tailored to specific data projects.

There are two basic AI models currently in use:

1. Discriminative AI – used for supervised machine learning (ML); it functions to separate the data points into different classes using probability estimates and identification of patterns (like image recognition and other computer vision tasks in sciences, including parasitology)
2. Generative AI – whose role is to generate new data points, as unsupervised ML. The computer ‘understands’ how the data are distributed and can create new data points and complex relationships between them with a great degree of freedom (like ChatGPT, self-driving cars or new drug design).

Lab diagnostics (including parasitology) utilizes discriminative AI models; they apply defined algorithms but also continuously optimize them to improve performance with experience. This is done through reinforcement learning (‘trial and error’) and can be accelerated by transfer learning (applying knowledge in one area to a different but related task).

AI systems evolved even further, beyond basic ML, to ‘deep learning’, which utilizes artificial neural networks (ANNs), modelled after the human brain. These networks are an array of interconnected nodes relaying signals between various layers of data and algorithms, helping to find connections and extract meaning from millions of data points. Convolutional neural networks (CNNs) are a type of ANN that are particularly suited for processing images; they excel in image classification and object detection tasks and can adapt over time. Trained on vast datasets from labelled parasite images, they can accurately detect and categorize images into predefined classes and basically play the role of an AI microscopist! [40,41].

An example of CNN-powered deep ML would be the utilization of computer vision for parasitology diagnostics including malaria, urinary schistosomiasis or interpretation of faecal smears. Digital scanning for organic shapes and training to recognize defined subsets (e.g. protozoan genera and species) aim to mimic the actions of a human microscopist; this requires application of deep learning-based CNN models. However, not all specimens present comparable levels of challenges. Blood or urine represents a relatively homogenous and fluid matrix, but the most common clinical sample, such as human stool, poses a far greater technical barrier as a complicated/heterogenous matrix, made of debris, food content, plant material and other microbiota. Therefore, many proof-of-concept studies, although encouraging, have not yet been fully validated and approved for diagnostic use on human stool specimens.

An initial clinical validation of AI detection of protozoa in trichrome-stained stools was carried out by Mathison *et al*. [42]. The goal of this study was to develop and verify a sensitive CNN AI model for screening out negative trichrome slides, while flagging potential parasites for manual confirmation.

The initial CNN training process was followed by clinical validation of the model by testing a set of unique positive slides (containing intestinal protozoa) and a set of negative slides, both derived from patient stool specimens. Accuracy (slide-level agreement with microscopy, i.e. parasite present or absent) was calculated as 98.88% (for positive agreement) and 98.11% (for negative agreement); this was deemed a robust enough tool for augmenting conventional microscopy (but not to replace it).

A few AI technologies have already been commercialized. One of these, the SightDx P1 platform for malaria diagnostics, showed a sensitivity of 97.03% and a specificity of 96.33% [43]. Others, like Techcyte Inc. (Orem, UT), AI software coupled with a Hamamatsu NanoZoomer 360 Digital slide scanner, have been evaluated at Mayo Clinic and found to be useful as an *augmentation* of the current manual method and workflow [44].

One step further – combining AI with automation – is Orienter Model FA280, a fully automatic digital faeces analyser for ova and parasite detection evaluated in Thailand in 2024 [45]. Interestingly, its performance significantly improved with the introduction of ‘user audit’ – a skilled microscopist review of AI data; this led the authors to conclude that ‘analyzer cannot fully replace a skilled operator. It still requires an experienced or well-trained laboratory technician to evaluate the digital microscope images to obtain accurate results and avoid misdiagnosing parasitic infections’.

The potential for AI-supported digital microscopy to be useful in primary healthcare may address the lack of technical expertise on site and improve access to diagnostics; a scoping review was initiated in January 2023 [46].

Beyond enhancing diagnostic parasitology, AI has the potential to do much more: it can fuel antiparasitic drug discovery, facilitate disease control through predictive modelling of outbreaks and support molecular research, to name only a few [47,48]. It is quite clear that the potential for collaboration between AI and human expertise exists and even flourishes, as it strays away from the traditional master–servant relationship between computers and users.

The issue, however, is complex and still subject to controversy. Focusing specifically on parasitology and its relationship with AI, one thing is clear: although significant strides have been made in the field, there are still many challenges that most of us do not appreciate. One of them is that parasitology, just as mycology, is heavily image-dependent, and quality is a huge problem.

As with any ML process, input of enormous numbers of high-quality microscopic details is essential. This is particularly difficult since training datasets may be inadequate for rare or unusual parasites. Even for commonly encountered organisms, their visual appearance may be affected by diversity within the species, individual morphologies and life stages. As mentioned before, for some biological material, heterogeneity of matrix, such as debris, food, plant material and other microbiota, creates multilevel challenges for AI training and validation process and algorithms.

Organism-derived parameters may also change in different host populations, and mundane factors such as sample background and lighting variability, or artefacts obscuring parasite forms, can significantly affect digital image perception.

However, the factor probably most relevant to all upcoming diagnostic challenges is an impending influx of uncommon or unexpected parasites. Relative rarity of some of these species leads to training of AI models on imbalanced datasets and results in biassed predictions, which can then self-propagate. Preventing this requires specialized approaches, such as deep learning models – including CNN-based transfer learning and data augmentation – as well as ongoing tracking of performance [48,49], which we are just now learning to appreciate.

And so, the success of AI in parasitology still depends on ‘biological’ neuronal connections – human expertise – to not only develop safe ML modalities but also to constantly monitor and adjust their functioning. This entails loading billions of data points, as well as balancing existing datasets to avoid learning bias. This job still requires content experts: people whose expertise takes years to develop… but who are now leaving and taking their knowledge banks with them.

We are entering a new diagnostic paradigm, akin to the arrival of self-driving cars, with great enthusiasm – but, at the same time, lesser understanding of what may happen just down the road. The AI solution as a remedy for labour shortage is indeed close, but still just beyond our reach, as we are preparing for that key transition phase.

Unfortunately, these preparations are fraught with challenges.

**Fig. 4.**
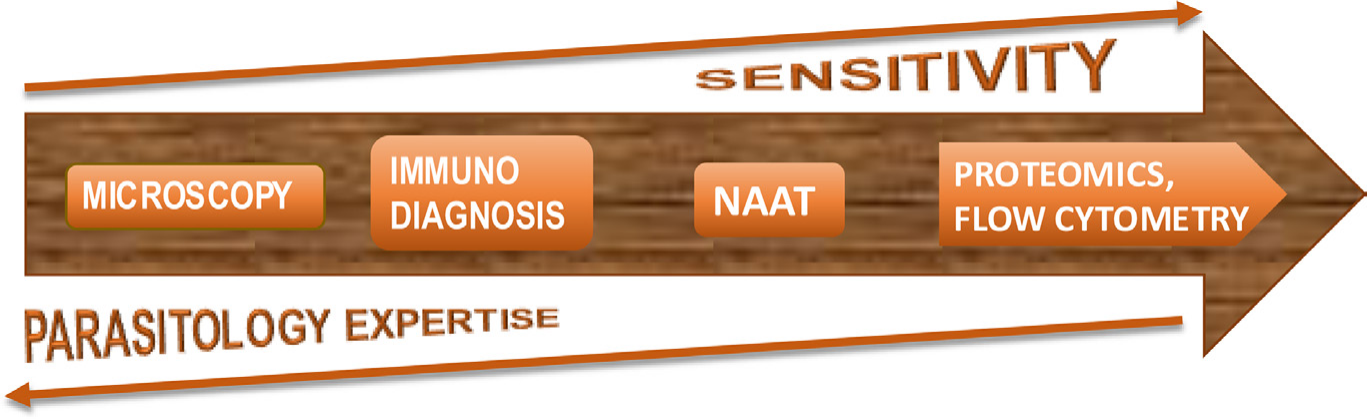
Evolution of diagnostic tools and approaches in clinical parasitology.

Driven by economic and sociological considerations, we are moving away from subject knowledge and shifting towards ‘method expertise’ (Fig. 4), hoping for easy hands-off solutions.

This, however, has consequences. Increased reliance on molecular detection methodologies and AI is a double-edged sword: on one hand, it saves time (if not money) and addresses the shortage of a specialized workforce, but on the other hand, it creates progressive, widespread loss of skills and expertise in parasitology and, consequently, our ability to adjust to change.

As our focus shifts away from the broader, principle-based approaches, we are losing the adaptability needed to handle Nature’s unexpected. Such adaptability draws on experience, just as deep ML relies on transfer learning. We are depleting national and international human knowledge banks, as they are gradually replaced by specialized technical prowess.

As a result, as we abrogate our responsibility to learn and maintain the basics, navigation of our shifting challenges will become even more difficult. This will ultimately impact patient care when diseases do not follow expected patterns. Moreover, in a vicious circle, it will also affect the development of new, improved generations of AI – a process hindered by our dwindling ability to load, label and monitor new data and algorithms.

Moving forward towards change does not always constitute true progress. Ignoring or disrespecting the past denies us the tools we need to critically analyse the present and notice patterns that might otherwise be invisible. These basic tools provide us with a crucial perspective to understand and anticipate changes and solve future problems – true for parasitology as well as many other disciplines.

Fortunately, just as with climate change, it is still not too late to adjust course and start applying multilayered solutions to multilayered challenges. It may just mean going a little ‘backwards’ and nurturing and supporting traditional expertise, hand in hand with advanced molecular methodologies and in harmony with AI.

## Abbreviations

AI: artificial intelligence
ANNs: artificial neural networks
CNNs: convolutional neural networks
GI: gastrointestinal
LFA: lateral flow assay
ML: machine learning
NAAT: nucleic acid amplification tests
